# Relationships between episodic memory performance prediction and sociodemographic variables among healthy older adults

**DOI:** 10.1590/S1980-57642015DN91000008

**Published:** 2015

**Authors:** Glaucia Martins de Oliveira, Meire Cachioni, Deusivania Falcão, Samila Batistoni, Andrea Lopes, Vanessa Guimarães, Thais Bento Lima-Silva, Anita Liberalesso Neri, Mônica Sanches Yassuda

**Affiliations:** 1 Degree in Gerontology – Escola de Artes, Ciências e Humanidades da Universidade de São Paulo.; 2 Associate Professor in Gerontology – Escola de Artes, Ciências e Humanidades da Universidade de São Paulo.; 3 Assistant Professor in Gerontology - Escola de Artes, Ciências e Humanidades da Universidade de São Paulo.; 4 Degree in Gerontology – Escola de Artes, Ciências e Humanidades da Universidade de São Paulo. Masters in Neurology from the Department of Neurology, Faculdade de Medicina da Universidade de São Paulo.; 5 Full Professor at UNICAMP School of Education.

**Keywords:** memory, memory prediction, metamemory, older adults

## Abstract

Previous studies have suggested that performance prediction, an aspect of metamemory, may be associated with objective performance on memory tasks.

**Objective:**

The objective of the study was to describe memory prediction before performing an episodic memory task, in community-dwelling older adults, stratified by sex, age group and educational level. Additionally, the association between predicted and objective performance on a memory task was investigated.

**Methods:**

The study was based on data from 359 participants in the FIBRA study carried out at Ermelino Matarazzo, São Paulo. Memory prediction was assessed by posing the question: "If someone showed you a sheet with drawings of 10 pictures to observe for 30 seconds, how many pictures do you think you could remember without seeing the sheet?". Memory performance was assessed by the memorization of 10 black and white pictures from the Brief Cognitive Screening Battery (BCSB).

**Results:**

No differences were found between men and women, nor for age group and educational level, in memory performance prediction before carrying out the memory task. There was a modest association (rho=0.11, p=0.041) between memory prediction and performance in immediate memory. On multivariate linear regression analyses, memory performance prediction was moderately significantly associated with immediate memory (p=0.061).

**Conclusion:**

In this study, sociodemographic variables did not influence memory prediction, which was only modestly associated with immediate memory on the Brief Cognitive Screening Battery (BCSB).

## Abstract (pt)

Estudos prévios sugerem que a previsão de desempenho, um dos aspectos da metamemória, pode estar associada ao desempenho em tarefas de memória.

**Objetivo:**

Objetivou-se descrever a previsão de desempenho antes da realização de uma tarefa de memória episódica em idosos residentes na comunidade, divididos por sexo, faixas de idade e escolaridade. Adicionalmente, objetivou-se estudar a associação entre a previsão de desempenho com o desempenho objetivo em tarefa de memória.

**Métodos:**

Foram utilizados os dados de 359 idosos participantes do estudo FIBRA realizado em Ermelino Matarazzo, São Paulo. Foi utilizada uma questão referente à previsão de desempenho: "Se alguém lhe mostrasse uma folha com desenhos de 10 figuras para observar por 30 segundos, quantas figuras o/a senhor/a acha que conseguiria se lembrar em seguida sem ver a folha?". O desempenho de memória foi avaliado pela memorização de 10 figuras em preto e branco da Bateria Breve de Rastreio Cognitivo (BBRC).

**Resultados:**

Não foram encontradas diferenças entre homens e mulheres, entre faixas etárias ou de escolaridade para a previsão de desempenho antes da realização de tarefa de memória. Houve associação modesta (rho=0,11, p=0,041) entre a previsão de desempenho e o desempenho em memória imediata. Na análise de regressão linear multivariada a previsão de desempenho associou-se de forma moderatemente significante com a memória imediata (p=0,061).

**Conclusão:**

Neste estudo observou-se que as variáveis sociodemográficas não influenciaram a previsão de desempenho que esteve associada de forma modesta com a memória imediata na Bateria Breve de Rastreio Cognitivo (BBRC).

## INTRODUCTION

Some cognitive functions tend to decline with age, such as episodic and working memory, executive functions and attention.^1^ However, some aspects of cognition, such as metamemory, remain less well studied in aging.

The term "metamemory" originally referred to a broad array of knowledge which people held about memory.^2^ The concept now also encompasses beliefs, such as self-efficacy, performance prediction and emotions in memory. Metamemory is especially relevant in Gerontology, since it is held that beliefs about memory can affect performance of older adults on memory tasks.^3-5^ Negative beliefs about memory are thought to have a deleterious impact on the use of strategies, on effort dedicated to the task and goal-setting, all of which can be regarded as moderating variables of the relationship between beliefs and memory performance.^6^

Metamemory can be studied by means of performance prediction. Under this paradigm, participants are asked to make predictions about their performance before performing memory task. Performance prediction is thought to involve an assessment of the difficulty level of the proposed task, together with an assessment of one's own ability to perform that task.^5^

Studies on performance prediction in memory tasks have produced mixed results, but tend to indicate that elderly overestimate their performance - not as a result of over confidence, but because they tend to under estimate the difficulty level of the task.^5,7^

In Brazil, Yassuda et al.^8^ assessed the validity of the Portuguese version of a metamemory questionnaire, the Metamemory in Adulthood Questionnaire (MIA) and also of a self-efficacy questionnaire, the Memory Self-Efficacy Questionnaire (MSEQ), in 33 younger and 27 older healthy Brazilian adults. The results of the analyses suggested that the Portuguese versions of these instruments exhibit good psychometric characteristics and are promising for research use in Brazil.

The relationship between performance prediction and objective performance on memory tasks may be influenced by the low level of schooling often found among elderly in the Brazilian milieu. Therefore, the objective of the present study was to describe memory prediction before performing a visual episodic memory task, in a sample of older adults from Ermelino Matarazzo, participants of the FIBRA study, stratified by sex, age group and educational level. Additionally, the association between predicted and objective performance on an episodic memory task was investigated.

## METHODS

**Participants.** This study was based on data from 359 participants of the population-based study "Frailty profiles in Brazilian elderly", conducted by the Fibra Network, UNICAMP, in response to Public call MCT-CNPq/MS-SCTIE-DECIT - no. 17/2006. A total of 384 elderly residents of Ermelino Matarazzo were interviewed between July 2008 and June 2009. The present study included all participants with complete data for the variables of interest (N=359). Participants scoring below the cut-off score on the Mini-Mental State Exam (MMSE) were not excluded.^9^ Further information on the methods employed in the FIBRA study is available in Neri et al.^10^

**Instruments.** This study was approved by the Research Ethics Committee of the School of Medical Sciences of the State University of Campinas, under report number 208/2007. All participants completed an extensive protocol, during a single session, which included sociodemographic variables, health-related variables, anthropometric measures, psychosocial variables and variables on frailty criteria.

Participants completed the MMSE and answered the following question about memory performance prediction: "If someone showed you a sheet with drawings of 10 pictures to observe for 30 seconds, how many pictures do you think you could remember without seeing the sheet?". The participants then carried out a memory task from the Brief Cognitive Screening Battery.^11^

The task consisted of naming and memorization of 10 common black and white drawings. The figures were named by the subject (Naming), who was then asked to recall each drawing immediately, without having been told the figures had to be memorized (Incidental Memory). Subsequently, the figures were displayed again and the subject asked to memorize them for 30 seconds for further recall (Immediate Memory). The procedure was then repeated (Learning). After performing other tasks for around 5 minutes, the subject was asked to evoke the figures shown previously (Delayed Memory). Finally, the 10 figures, mixed with another 10 distractor figures, were redisplayed and the participant asked to recognize those figures displayed originally (Recognition). Scoring on these tests ranges from 0 to 10 points.

For the present study, data related to performance on memory and performance prediction tests were analyzed. Sociodemographic data were used as independent variables and to characterize the sample.

**Data analyses.** The Chi-square test was employed to compare the categorical variables between groups. The absence of a normal distribution for the continuous variables dictated the use of non-parametric tests, namely, the Mann-Whitney and Kruskall-Wallis U-tests. When a p-value <0.05was determined on the Kruskall-Wallis test, the Multiple Comparisons z values test was applied. Spearman Correlation analyses were also carried out.

For analysis of the relationship between predicted and objective performance on memory tests in the presence of sociodemographic variables, linear regression analysis was carried out with the multivariate model using Stepwise Forward variable selection criteria, i.e. from the most simple to most complex model. The variables yielding p<0.10 on univariate regression analyses were included in the final multiple models. The variables sex, age, schooling, family income in minimum wages and performance prediction were included in the models as independent variables whereas the cognitive variables from the BCSB were used as dependent variables in separate models (MMSE, Naming, Incidental Memory, Immediate Memory, Learning, Delayed Memory and Recognition).

The data were keyed into Version 3.1 of the Epidata Program. All statistical analyses were performed using the SPSS v.17.0 and Statistica v. 7.0 software packages. The level of significance adopted for the statistical tests was 5%, i.e. a p-value<0.05.

## RESULTS

The sample comprised predominantly female participants, aged 65-75 years, married or in common law union and educated to primary school level (Table 1).

**Table 1:** Data characterizing the sample (n=359).

Variables		N	%
Sex	Male	120	33.43
	Female	239	66.57
Age groups	65-69	138	38.44
	70-74	115	32.03
	75-79	63	17.55
	80 or over	43	11.98
	Mean (SD)	72.16	5.65
	Median	71.00	
	Minimum – Maximum	71.00-92.00	
Marital status	Single	29	8.08
	Married/Stable union	178	49.58
	Divorced, legally separated	26	7.24
	Widow(er)	126	35.10
Schooling (in years)	Illiterates	62	17.27
	From 1 to 4 years	225	62.67
	From 5 to 8 years	59	16.43
	From 9 to 11 years	7	1.95
	12 years or more	6	1.67
	Mean (SD)	3.46	2.81
	Median	4.00	
	Minimum – Maximum	21.00	
Personal income	Up to 1.0 MW	144	40.11
	From 1.1 to 3.0 MWs	153	42.62
	From 3.1 to 5.0 MWs	40	11.14
	From 5.1 to 10.0 MWs	8	2.23
	Over 10 MWs	4	1.11
	Not informed	10	2.79
Family income	Up to 1.0 MW	26	7.24
	From 1.1 to 3.0 MWs	164	45.68
	From 3.1 to 5.0 MWs	74	20.61
	From 5.1 to 10.0 MWs	32	8.91
	Over 10 MWs	10	2.79
	Not informed	53	14.76
Social welfare benefits	None	42	11.70
	Retired	204	56.82
	Pensioners	65	18.11
	Retired and Pensioners	48	13.37

MW: minimum wage.

Participants predicted recall of an average of five figures (SD=2.3) if asked to memorize 10 figures (Table 2). Participants scored an average of 23.90 (SD=3.56) on the MMSE and 7.49 (SD=1.97) on the Delayed Memory task. No significant difference in performance prediction was found between men and women. Table 2 reveals that men performed better than women on the MMSE (24.75(M) vs. 23.48 (W) p<0.001).

**Table 2:** Mean and standard deviations for metamemory and cognitive performance variables for total sample and for men and women.

Variable	Mean	SD±	Minimum	Median	Maximum	Men (n=120)		Women (n=239)	p-value
						Mean SD		Mean SD	
Predicted number of figures recalled									
Total	5.04	2.31	0.00	5.00	10.00	4.95 (1.99)		5.09 (2.46)	0.984
Total scores on tests									
MMSE	23.90	3.56	9.00	24.00	30.00	24.75 (3.51)		23.48 (3.51)	<0.001
Naming	9.70	1.21	0.00	10.00	10.00	9.56 (1.57)		9.77 (0.98)	0.275
Incidental	5.66	1.47	0.00	6.00	10.00	5.37 (1.41)		5.80 (1.48)	0.017
Immediate	7.57	1.59	0.00	8.00	10.00	7.21 (1.70)		7.75 (1.50)	0.003
Learning	8.25	1.61	0.00	8.00	10.00	8.12 (1.58)		8.32 (1.62)	0.177
Delayed	7.49	1.97	0.00	8.00	10.00	7.38 (1.83)		7.54 (2.04)	0.183
Recognition	9.39	1.08	0.00	10.00	10.00	9.40 (1.05)		9.38 (1.10)	0.801

MMSE: Mini-mental State Exam.

No significant difference in performance prediction was found for age or schooling (Table 3). Younger and more educated participants had better cognitive performance.

**Table 3:** Mean and standard deviations for metamemory and cognitive performance variables among elderly from different age and schooling groups.

**Variables**	**Age groups**											
	**65-69**			**70-74 **			**75-79 **			**80 or over **		**p-value**
	**Mean**	**SD±**		**Mean**	**SD±**		**Mean**	**SD±**		**Mean**	**SD±**	
**Predicted number of figures recalled**												
Total	4.80	2.14		5.24	2.35		4.92	2.22		5.47	2,77	0.277
**Total scores on tests:**												
MMSE	25.18	2.83		23.91	3.41		23.11	3.06		20.93	4,58	**<0.001**
Naming	9.94	0.34		9.56	1.46		9.65	1.36		9.36	1,83	**0.002**
Incidental	6.07	1.30		5.58	1.46		5.44	1.32		4.81	1,81	**<0.001**
Immediate	8.10	1.19		7.43	1.56		7.40	1.70		6.45	1,93	**<0.001**
Learning	8.72	1.07		8.17	1.71		8.08	1.56		7.17	2,21	**<0.001**
Delayed	8.12	1.43		7.52	1.87		7.11	1.88		5.88	2,79	**<0.001**
Recognition	9.67	0.67		9.32	1.28		9.30	0.87		8.76	1,49	**<0.001**
	**Schooling**											
	**Illiterates**				**From 1 to 4 years**			**5 years or more**				**p-value**
	**Mean**	**SD±**			**Mean**	**SD±**		**Mean**			**SD±**	
**Predicted number of figures recalled**												
Total	5.31	2.91			4.96	2.25		5.07			1.88	0.836
**Total scores on tests**												
MMSE	20.77	3.96			24.48	2.99		24.78			3.42	**<0.001**
Naming	9.20	2.00			9.80	0.98		9.81			0.85	**<0.001**
Incidental	5.38	1.85			5.71	1.37		5.74			1.41	0.460
Immediate	7.15	1.85			7.61	1.59		7.81			1.23	0.137
Learning	7.70	1.99			8.28	1.55		8.60			1.31	**0.020**
Delayed	6.89	2.67			7.58	1.78		7.71			1.77	0.323
Recognition	9.08	1.16			9.43	1.07		9.51			1.01	**0.015**

Age groups (MMSE: 65-69≠70-74, 65-69≠75-79, 65-69≠80 or over, 70-74≠80 or over. Incidental: 65-69≠75-79, 65-69≠80 or over. Immediate: 65-69≠70-74, 65-69≠75 79, 65-69≠80 or over, 70- 74≠80 or over. Learning: 65-69≠80 or over, 70-74≠80 or over. Delayed Recall: 65 69≠75-79, 65-69≠80 or over, 70-74≠80 or over. Recognition: 65-69≠75-79, 65 69≠80 or over. Schooling (MMSE: Illiterates≠1 to 4 years, Illiterates≠5 years or more. Learning: Illiterates≠5 years or more. Naming: Illiteratess≠1 to 4 years.

Table 4 depicts correlations, revealing a significant association among the cognitive variables. The performance prediction variable was moderately but significantly associated with Immediate Memory.

**Table 4:** Correlation matrix between performance prediction and cognitive variables.

	Performance prediction	MMSE	Naming	Incidental	Immediate	Learning	Delayed memory
MMSE	rho=-0.03 p=0.601						
Naming	rho=-0.03 p=0.621	0.26 <0.001					
Incidental	rho=0.10 p=0.053	0.29 <0.001	0.16 0.003				
Immediate	rho=0.11 p=0.041	0.30 <0.001	0.22 <0.001	0.54 <0.001			
Learning	rho=0.02 p=0.660	0.32 <0.001	0.20 <0.001	0.42 <0.001	0.58 <0.001		
Delayed	rho=0.04 p=0.450	0.31 <0.001	0.18 <0.001	0.44 <0.001	0.55 <0.001	0.69 <0.001	
Recognition	rho=0.08 p=0.123	0.34 <0.001	0.27 <0.001	0.31 <0.001	0.34 <0.001	0.31 <0.001	0.33 <0.001

rho (Spearman’s correlation coefficient) and p (p-value).

The multivariate regression analysis (Table 5) revealed that the cognitive variables were influenced by schooling, age and sex. Performance prediction had no significant influence on cognitive variables but was moderately associated with Immediate Memory.

**Table 5:** Multiple linear regression analyses for sex, age, schooling, income and performance prediction as independent variables. Variable selection criteria: Stepwise Forward Modeling (p<0.10).

Performance	Independent variables	B	Standard error	p-value
MMSE	Age	–0.217	0.030	**<0.001**
	Schooling	0.355	0.059	<0.001
	Sex	–1.076	0.349	**0.002**
Naming	Age	–0.032	0.012	**0.005**
	Schooling	0.060	0.023	**0.010**
	Sex	0.250	0.135	0.065
Incidental	Age	–0.068	0.013	**<0.001**
	Sex	0.414	0.159	**0.009**
Immediate	Age	–0.087	0.014	**<0.001**
	Sex	0.618	0.168	<0.001
	Income	0.000	0.000	**0.003**
	Performance prediction	0.066	0.035	0.061
Learning	Age	–0.080	0.015	**<0.001**
	Schooling	0.068	0.029	**0.020**
Delayed	Age	–0.126	0.018	**<0.001**
Recognition	Age	–0.042	0.010	**<0.001**
	Schooling	0.046	0.020	**0.020**

Reference for the sex variable is male gender.

## DISCUSSION

In the present study, no influence of sex, age or schooling on performance prediction was found. Performance prediction was moderately associated with Immediate Memory.

In contrast with the present findings, a previous study detected an association between performance prediction and sex. Hertzog, Dixon and Hultsch^12^ reported that women showed a significant increase in their sequential performance predictions, i.e. they were able to monitor their performance and revise predictions more effectively than men.

In the present study, older participants did not predict lower performance, as might be expected given age-related decline in episodic memory. Previous studies have reported the influence of age on performance prediction but included younger adults, children and older adults in their samples.^12-14^

With regard to schooling, elderly with higher educational level, who generally perform better on memory tasks, can be expected to have superior performance prediction. However, this expectation was not confirmed by the present data. Additionally, no association was found between predicted and objective performance on memory tasks, in contrast with previous international studies. In the present study, analysis of the distribution of scores for the performance prediction variable showed that a significant number of participants gave an intermediate prediction value (5). It is possible that faced with the difficulty of providing an estimate on future performance, participants chose a mid-point on the scale, which may partially explain the disparities with previous studies. The present study should encourage other researchers to investigate this aspect of metamemory and its relationship with performance on episodic memory tasks.

Myths and stereotypes associated with aging can potentially exert an influence on cognitive performance of elderly individuals. Future studies should further investigate the variables associated with the metamemory construct, which has been little investigated in Brazil's elderly population.

To conclude, sociodemographic variables did not influence predicted memory performance, which was only modestly associated with immediate memory on the BCSB.
